# TIM-3 Expression Level on AML Blasts Correlates With Presence of Core Binding Factor Translocations Rather Than Clinical Outcomes

**DOI:** 10.3389/fonc.2022.879471

**Published:** 2022-04-14

**Authors:** Jian Hong, Leiming Xia, Zhenqi Huang, Xiaodong Yuan, Xinglin Liang, Jifei Dai, Zhonghui Wu, Li Liang, Min Ruan, Zhangbiao Long, Xin Cheng, Xiaowen Chen, Jing Ni, Jian Ge, Qingsheng Li, Qingshu Zeng, Ruixiang Xia, Yi Wang, Mingzhen Yang

**Affiliations:** ^1^ Department of Hematology, The First Affiliated Hospital of Anhui Medical University, Hefei, China; ^2^ Department of Hematology, The Forth Affiliated Hospital of Anhui Medical University, Hefei, China; ^3^ Division of Life Sciences and Medicine, Department of Organ Transplantation Center, Transplant and Immunology Laboratory, The First Affiliated Hospital of University of Science and Technology of China (USTC), University of Science and Technology of China, Hefei, China; ^4^ Department of Oncology, The Third Affiliated Hospital of Anhui Medical University, Hefei, China

**Keywords:** TIM-3, acute myeloid leukemia, core binding factor translocation, prognosis, flow cytometry

## Abstract

**Background:**

T-cell immunoglobulin and mucin domain-containing molecule 3 (TIM-3) expresses on leukemic stem and progenitor populations of non-M3 acute myeloid leukemia (AML) as well as T lymphocytes. TIM-3 is thought to be involved in the self-renewal of leukemic stem cells and the immune escape of AML cells, however its correlation with AML prognosis is still controversial and worthy of further investigation.

**Methods:**

we simultaneously assessed TIM-3 expression levels of leukemic blasts and T lymphocytes in the bone marrow of *de novo* AML patients using flow cytometry. The correlations of TIM-3 expression between leukemic blasts and T lymphocytes and the correlations of TIM-3 expression with various patient parameters were analyzed. In addition, the Cancer Genome Atlas (TCGA) data of AML patients were acquired and analyzed to verify the results.

**Results:**

TIM-3 expression of CD34^+^ leukemic blasts (R^2 ^= 0.95, p<0.0001) and CD34^+^CD38^-^ leukemic stem cells (R^2 ^= 0.75, p<0.0001) were significantly and positively correlated with that of the whole population of leukemic blasts. In addition, TIM-3 expression level of leukemic blasts correlated significantly and positively with that of CD8^+^ (R^2 ^= 0.44, *p*<0.0001) and CD4^+^ (R^2 ^= 0.16, *p*=0.0181) lymphocytes, and higher TIM-3 expression of leukemic blasts was significantly associated with a greater proportion of peripheral CD8^+^ T lymphocytes (R^2 ^= 0.24, *p*=0.0092), indicating that TIM-3 on leukemic blasts might alter adaptive immunity of AML patients. Regarding clinical data, the presence of core binding factor (CBF) translocations was significantly correlated with higher TIM-3 expression of leukemic blasts (CBF versus non-CBF, median 22.78% versus 1.28%, *p*=0.0012), while TIM-3 expression levels of leukemic blasts were not significantly associated with the remission status after induction chemotherapy (*p*=0.9799), overall survival (*p*=0.4201) or event-free survival (*p*=0.9873). Similar to our results, TCGA data showed that patients with CBF translocations had significantly higher mRNA expression level of HAVCR2 (the gene encoding TIM-3) (median, 9.81 versus 8.69, *p*<0.0001), and as all patients in the cohort were divided into two groups based on the median HAVCR2 expression level, 5-year overall survivals were not significantly different (low versus high, 24.95% versus 24.54%, *p*=0.6660).

**Conclusion:**

TIM-3 expression level on AML blasts correlates with presence of CBF translocations rather than clinical outcomes.

## Introduction

T-cell immunoglobulin and mucin domain-containing molecule 3 (TIM-3) is a membrane protein which was discovered initially on CD4^+^ T helper 1 (Th1) and CD8^+^ T cytotoxic 1 cells (1), and later on a variety of other cell types including regulatory T (Treg) cells (2), dendritic cells (3), natural killer (NK) cells (4), myeloid cells (5) and mast cells (6). TIM-3 is generally considered as a negative regulator of immune system (7), and has been investigated as a blockade target for immunotherapy of cancer in pre-clinical and clinical settings (8–10).

Interestingly, TIM-3 also expresses on leukemic stem and progenitor populations of non-M3 acute myeloid leukemia (AML) and associated with leukemic transformation of preleukemic diseases, such as myelodysplastic syndrome (MDS) and myeloproliferative neoplasm (11). TIM-3 and its ligand, galectin-9 (Gal-9), constitute an autocrine loop which drives the self-renewal of AML stem cells by activating the nuclear factor-κB (NF-κB) and β-catenin pathways (11). Besides, both TIM-3 and Gal-9 can be released in a free soluble form and involved in the immune escape of AML cells (12).

TIM-3 expression on immune cells has been reported to be associated with disease activity and prognosis in multiple types of cancers, e.g. AML (13), pediatric B-precursor acute lymphoid leukemia (14), lung cancer (2, 4). In general, higher TIM-3 expression was related to poorer prognosis. Additionally, the relation between TIM-3 expression on AML blasts and prognosis of AML was also investigated and contradictory data were published in the literature. Darwish et al. and Kamal et al. both reported TIM-3 as a biomarker of poor prognosis in AML, while Xu et al. reported that increased TIM-3 expression correlated with low-risk group and higher complete remission rate in newly diagnosed non-M3 AML patients (15–17).

According to the data mentioned above, TIM-3 expression on T cells and AML blasts are both related to prognosis of AML, and there might be an association between TIM-3 expression on T cells and AML blasts. To our knowledge, this issue has not been well investigated and published in the literature. In this study, we assessed simultaneously the TIM-3 expression on T cells and AML blasts in patients with non-M3 *de novo* AML using flow cytometry, and investigated its correlation with clinical outcomes, as well as other clinical parameters, including French-American-British (FAB) classifications, genetic abnormalities and risk stratifications. Furthermore, the Cancer Genome Atlas (TCGA) data for AML were obtained and analyzed to validate the impact of TIM-3 expression on prognosis of non-M3 AML patients.

## Materials and Methods

### Patients

A total of 34 patients diagnosed with *de novo* AML (except for M3) were recruited from the Department of Hematology, the First Affiliated Hospital of Anhui Medical University (Hefei, China) between July 2018 and May 2020. The demographic and clinical data of these patients were shown in. The diagnosis and classification of these patients were based on the revised FAB classification and the 2016 World Health Organization (WHO) criteria. Smears of bone marrow aspirates were stained with Wright−Giemsa stain. Immunophenotyping analyses of leukemic cells were applied according to the “EGIL recommendations”. Eight important fusion genes, including PML-RARα, AML1-ETO, CBFβ-MYH11, BCR-ABL, MLL-AF9, DEK-CAN, PLZF-RARα and NPM-MLF, were detected using reverse transcriptase-polymerase chain reaction (RT-PCR). Next-generation sequencing-based detection of somatic mutations were performed using bone marrow samples for a panel of 20 genes (ASXL1, BCOR, CEBPA, DNMT3A, EZH2, FLT3, GATA2, IDH1, IDH2, KIT, KRAS, MLL, NPM1, NRAS, PDGFRA, PHF6, RUNX1, TET2, TP53 and WT1). Karyotypic analyses were performed using conventional R-banding assay, following standard 24-hour unstimulated culture of bone marrow samples. Up to 20 cells were analyzed for clonal abnormalities according to the International System for Human Cytogenetic Nomenclature (ISCN 2005) guidelines. Patients were divided into low, intermediate and high risk groups according to the 2017 European LeukemiaNet (ELN) risk stratification by genetics. FLT3-ITD was detected using next-generation sequencing rather than PCR-based deoxyribonucleic acid analysis, therefore allelic ratio of FLT3-ITD could not be calculated and all cases of FLT3-ITD were considered as high allele ratio during ELN risk stratification.

Thirty-two Patients received induction chemotherapies and the other two patients were given supportive care. The details of chemotherapy regimens were listed in **Table 1**. The responsiveness to chemotherapy of all surviving patients (27 out of 32) was assessed by the end of the induction chemotherapy. The assessment included a full blood work up and a bone marrow examination. Patients were classified into responders and non-responders. Responders attained complete remission (CR) or complete remission with incomplete count recovery (CRi). CR was defined as absolute neutrophilic count >1,000/μl, a platelet count ≥100,000/μl and <5% bone marrow blasts in a normocellular marrow, with no evidence of extramedullary disease. CRi was defined as <5% bone marrow blasts and no evidence of extramedullary disease without achieving the criteria of CR. Overall survival (OS) was measured from the date of diagnosis to the date of death from any cause. Event-free survival (EFS) was measured from the date of diagnosis to the date of primary refractory disease, relapse, or death from any cause. Patients with minimal residual disease were not considered relapsed for EFS determination.

**Table 1:** Demographic and clinical data of AML patients.

Patient characteristics (n=34)	
Gender, male/female	19/15
Median age, years(range)	51 (23-67)
Median WBC, ×10^9^/L(range)	18.3 (0.5-242.3)
Median HB, g/L(range)	85.0 (34.0-127.0)
Median PLT, ×10^9^/L(range)	38.5 (3.0-522.0)
FAB	
M1	2
M2	19
M4	5
M5	8
Karyotype	
normal	14
t(8;21) or AML1-ETO	4
inv(16) or t(16;16) or CBFβ-MYH11	4
t(9;22) or BCR-ABL	2
t(9;11) or MLLT3-KMT2A	2
complex	2
others	3
no data	3
Gene mutations	
FLT3-ITD	6
FLT3-TKD	3
NPM1	6
TET2	4
DNMT3A	5
ASXL1	3
KIT	3
NRAS	5
KRAS	1
IDH1	4
IDH2	4
single-mutated CEBPA	2
double-mutated CEBPA	4
no data	1
Risk stratification	
low	16
intermediate	5
high	11
not available	2
Treatment	
IA or DA	26
decitabine-based chemotherapy	4
CAG or DAG	2
supportive care	2

WBC, white blood cell; Hb, hemoglobin; PLT, platelet; FAB, French-American-British classification; IA, idarubicin and cytarabine; DA, daunorubicin and cytarabine; CAG, cytarabine, aclarubicin and recombinant granulocyte colony stimulating factor; DAG, daunorubicin, cytarabine and recombinant granulocyte colony stimulating factor.

The present study was approved by the Ethical Committee of the First Affiliated Hospital of Anhui Medical University and performed in accordance with the Declaration of Helsinki.

### Flow Cytometric Analysis

Flow cytometry was performed on a Navios Flow Cytometer (Beckman Coulter) and analyzed using Kaluza Flow Cytometry Analysis Software (Beckman Coulter). Fluorescence-conjugated monoclonal antibodies (mAbs) against CD45 (B36294, Beckman coulter, USA), CD3 (IM2472, Beckman coulter, USA), CD8 [IM2469, (B36294, Beckman coulter, USA)], CD34 (IM2472, Beckman coulter, USA), CD38 (B92396, Beckman coulter, USA) and TIM-3 (12-3109-42, eBioscience, USA) were used to analyze the surface expression of TIM-3 on leukemic blasts and T cells in the bone marrow. The gating strategy of TIM-3 expression of leukemic blasts and T lymphocytes is shown in **Supplementary Figure 1**. Peripheral lymphocyte populations including CD3^+^ T cells (CD3^+^), CD8^+^ T cells (CD3^+^CD8^+^), CD4^+^ T cells (CD3^+^CD4^+^), B cells (CD3^-^CD19^+^), natural killer cells (CD3^-^CD16^+^ or CD3^-^CD56^+^) were assessed according to manufacturer’s protocol (Tongsheng Shidai, Beijing, or Beckman coulter, USA) (**Supplementary Figure 2**). Peripheral regulatory CD4^+^ T cells were labeled with fluorescence-conjugated mAbs against CD4, CD25 and CD127 (Beckman coulter, USA), and the percentage of CD4^+^ Treg cells was calculated as CD4^+^CD25^+^CD127^-^ cells in the CD4^+^ cell population (**Supplementary Figure 3**). The appropriate isotypes were used as negative controls. Red blood cells in the samples were removed with red blood cell lysis buffer. Th1/Th2/Th17 cytokines in the plasma were assessed using cytometric bead array human Th1/Th2/Th17 cytokine kit according to manufacturer’s protocol (BD Biosciences, USA).

### TCGA Dataset

The expression data (RNA Seq V2 RSEM) of HAVCR2, the gene encoding TIM-3, from the peripheral blood of a cohort of 200 AML patients in TCGA were downloaded together with corresponding clinical data *via* cBioPortal (http://www.cbioportal.org) (18–20). In total, 157 patients were diagnosed with non-M3 AML and had HAVCR2 expression level. Their data were analyzed in this study. Regarding the risk stratification, 154 patients could be categorized into low, intermediate and high risk groups according to the 2017 ELN risk stratification by genetics, and the presence of FLT3-ITD were all considered as high allele ratio.

### Statistical Analysis

Correlations of TIM-3 expression levels between different groups and TIM-3 expression level of leukemic blasts with other parameters were assessed using the spearman’s rank correlation coefficient, and the linear regression was additionally carried out. The Mann-Whitney U test was used to compare differences between two groups, and the Kruskal-Wallis test followed by Dunn’s *post hoc* test was used to compare differences between multiple groups.

The probabilities of OS and EFS were estimated by the Kaplan-Meier method and compared by the log-rank test. The end point of the last follow-up for all surviving patients was September 12^th^, 2021. For all analyses, *p*<0.05 was considered statistically significant. The GraphPad Prism 6 (La Jolla, CA, USA) was used for data analyses.

## Results

### TIM-3 Expression of CD8^+^ and CD4^+^ T Lymphocytes Correlated Positively With That of Leukemic Blasts

**Figure 1:**
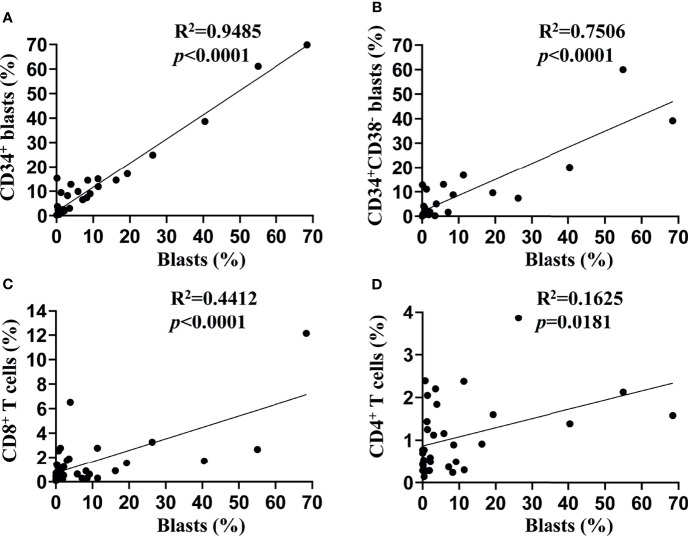
Associations of TIM-3 expression between subtypes of leukemic blasts and T lymphocytes. TIM-3 expression levels on the surface of leukemic blasts and T lymphocytes from bone marrow samples of 34 *de novo* AML patients were assessed using flow cytometry. Linear regression was performed to show associations of TIM-3 expression level of the whole population of leukemic blasts with that of CD34^+^ leukemic blasts **(A)**, CD34^+^CD38^-^ leukemic stem cells **(B)**, CD8^+^ T lymphocytes **(C)** and CD4^+^ T lymphocytes **(D)**.

We assessed the TIM-3 expression on leukemic blasts of 34 patients diagnosed with *de novo* AML (except for M3) using flow cytometry, and the percentage of TIM-3 expression ranged from 0 to 68.43%. Then, we compared the TIM-3 expression of the whole population of leukemic blasts with that of CD34^+^ leukemic blasts and CD34^+^CD38^-^ leukemic stem cells (LSCs). The results indicated that the TIM-3 expression of CD34^+^ leukemic blasts (R^2 ^= 0.95, *p*<0.0001) and CD34^+^CD38^-^ LSCs (R^2 ^= 0.75, *p*<0.0001) were significantly and positively correlated with that of the whole population of leukemic blasts (**Figures 1A, B**). Therefore, we decided to use the TIM-3 expression of the whole population of leukemic blasts for further analyses.

In addition, we assessed the TIM-3 expression of CD8^+^ and CD4^+^ T lymphocytes in the bone marrow of these AML patients, and both of them correlated positively with that of leukemic blasts (CD8^+^ T cells: R^2 ^= 0.44, *p*<0.0001; CD4^+^ T cells: R^2 ^= 0.16, *p*=0.0181) (**Figures 1C, D**).

### Higher TIM-3 Expression of Leukemic Blasts Was Significantly Associated With a Greater Proportion of CD8^+^ T Lymphocytes

**Figure 2:**
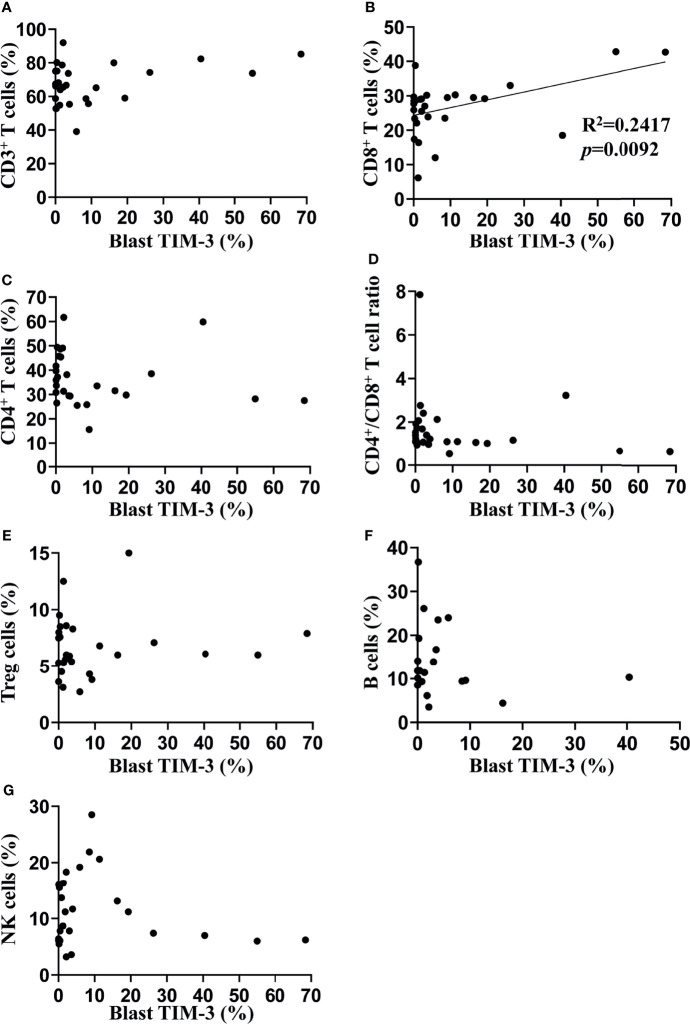
Associations of TIM-3 expression level of leukemic blasts with proportions of peripheral lymphocyte subtypes in AML patients. Proportions of multiple lymphocyte subsets were assessed in 27 out of 34 AML patients using flow cytometry (the percentage of B cells was only available in 20 patients). Linear regression was performed to determine associations of TIM-3 expression level of leukemic blasts with percentages of CD3^+^ T cells **(A)**, CD3^+^CD8^+^ T cells **(B)**, CD3^+^CD4^+^ T cells **(C)**, CD4^+^CD25^+^CD127^-^ Treg cells **(E)**, CD19^+^ B cells **(F)** and CD16^+^ or CD56^+^ NK cells **(G)**, as well as the ratio of CD4^+^/CD8^+^ T cells **(D)**.

In order to investigate the impact of TIM-3 expression of leukemic blasts on lymphocyte subtypes, we assessed the proportions of lymphocyte subtypes (CD8^+^ T cells, CD4^+^ T cells, CD4^+^ Treg cells, B cells and NK cells) and concentrations of helper T cell-1 (Th1)/Th2/Th17 cytokines (tumor necrosis factor-α, interferon-γ, interleukin-2 [IL-2], IL-4, IL-6, IL-10 and IL-17) in the peripheral blood of these AML patients. Results showed that the TIM-3 expression of leukemic blasts was significantly and positively associated with the proportion of CD8^+^ T lymphocytes (R^2 ^= 0.24, *p*=0.0092) but not other lymphocyte subtypes (**Figure 2**). Moreover, Th1/Th2/Th17 cytokine concentrations did not correlate with the TIM-3 expression of leukemic blasts (**Supplementary Figure 4**).

### TIM-3 Expression of Leukemic Blasts Was Associated With CBF Translocations But Not With Clinical Outcomes of AML Patients

**Figure 3:**
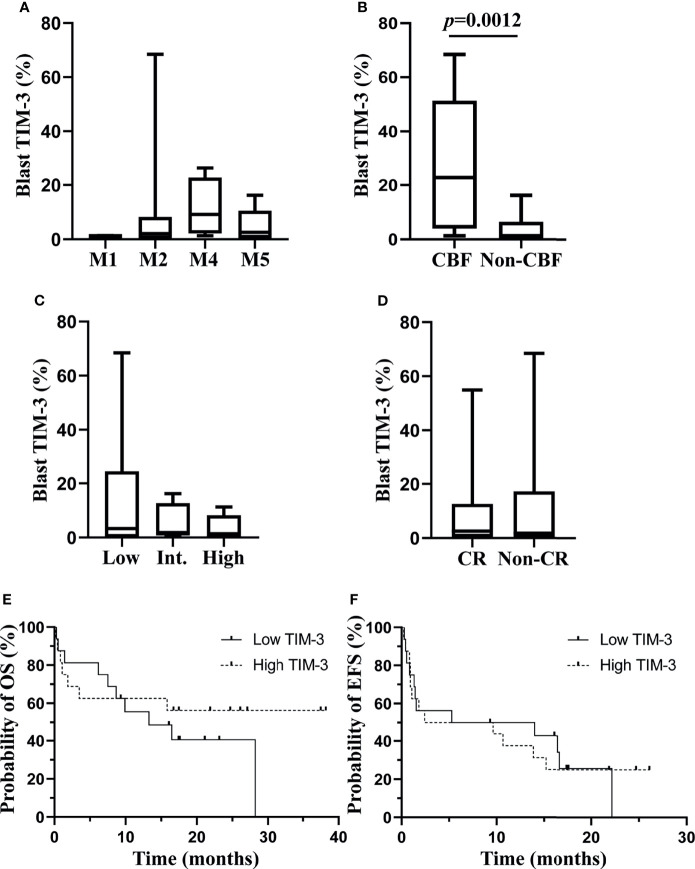
Associations of TIM-3 expression level of leukemic blasts with clinical parameters of AML patients. TIM-3 expression levels of leukemic blasts in FAB subtypes are shown in **(A)**. TIM-3 expression levels of leukemic blasts in patients with or without CBF translocations are shown in **(B)**. TIM-3 expression levels of leukemic blasts in ELN risk groups are shown in **(C)**. A total of 32 AML patients received the induction chemotherapy, and 18 patients were CR, 9 patients were non-CR and 5 patients died during the induction chemotherapy. TIM-3 expression levels of leukemic blasts in patients who achieved CR or not after induction chemotherapy are shown in **(D)**. These patients were divided into low and high TIM-3 groups based on the median TIM-3 expression level. Probabilities of OS and EFS of two groups are shown in **(E)** and **(F)**, respectively. **(A–D)** Box and whisker plots are used to show the data. Boxes represent the interquartile range, lines inside the boxes represent the median, and whiskers represent minimum and maximum values. CBF, core-binding factor; CR, complete remission; EFS, event-free survival; Int, intermediate; OS, overall survival.

We investigated the association of TIM-3 expression on leukemic blasts with multiple clinical characteristics of these AML patients. Data showed that TIM-3 expression levels on leukemic blasts were not significantly related to white blood cell count, hemoglobin concentration or platelet count at diagnosis (**Supplementary Figures 5A–C**). Regarding the FAB classification, a higher median TIM-3 expression level was observed in patients with M4, however the difference among subtypes was not statistically significant (*p*=0.2733) (**Figure 3A**). The relation of TIM-3 expression with genetic abnormalities was investigated as well, and results showed that patients with core binding factor (CBF) translocations, including AML1-ETO and CBFβ-MYH11 fusion genes, had significantly higher TIM-3 expression level compared to those without CBF translocations (median 22.78% versus 1.28%, *p*=0.0012) (**Figure 3B**). In addition, although the TIM-3 expression level of three ELN risk groups had no significant difference (*p*=0.7399), we might observe an accumulation of patients with higher TIM-3 expression level in the low risk group (**Figure 3C**).

With respect to the relation of TIM-3 expression with clinical outcomes of these AML patients, significantly different TIM-3 expression levels were not observed between patients who achieved CR or not after the induction chemotherapy (*p*=0.9799) (**Figure 3D**). Moreover, all patients were divided into low and high expression groups based on the median TIM-3 expression level, and the probabilities of 1-year OS (Low versus high, 55.56% [95% CI 75.88%-28.60%] versus 62.50% [95% CI 81.09%-34.86%], *p*=0.4201) and EFS (Low versus high, 50.00% [95% CI 24.52%-71.05%] versus 37.50% [95% CI 15.42%-59.77%], *p*=0.9873) did not differ significantly between two groups (**Figures 3E, F**).

### TCGA Dataset: The mRNA Expression of TIM-3 in the Peripheral Blood of Non-M3 AML Patients Was Not Associated With Their Clinical Outcomes

**Figure 4:**
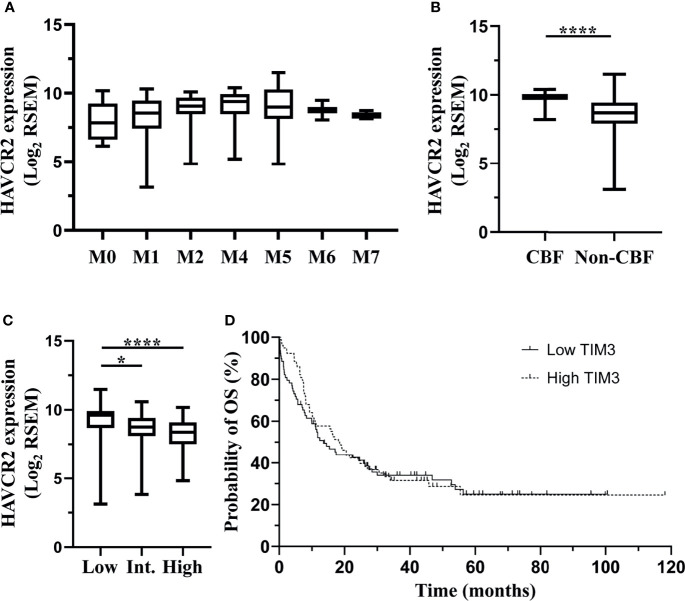
TCGA dataset: associations of HAVCR2 mRNA expression level in the bone marrow of non-M3 AML patients with clinical parameters. Data of a cohort of 200 AML patients from The TCGA were downloaded *via* cBioPortal. Among them, 157 patients who were diagnosed with non-M3 AML and had data of HAVCR2 mRNA expression (RNA Seq V2 RSEM, log2-transformed) were analyzed in this study. HAVCR2 is the gene encoding TIM-3. HAVCR2 expression levels in FAB subtypes are shown in **(A)**. HAVCR2 expression levels in patients with or without CBF translocations are shown in **(B)**. HAVCR2 expression levels in ELN risk groups are shown in **(C)**. Patients were divided into low and high TIM-3 groups based on the median HAVCR2 expression level. Probabilities of OS of two groups are shown in **(D)**. Statistical significance is displayed as *p<0.05, ****p< 0.0001. CBF, core-binding factor; Int, intermediate; OS, overall survival.

In order to validate the correlation of TIM-3 expression with clinical characteristics of AML patients, we performed an independent assessment of AML patients from TCGA database. Similar to our data, The mRNA expression level (RNA Seq V2 RSEM, log2) of HAVCR2, the gene encoding TIM-3, in the peripheral blood of AML patients was not significantly associated with white blood cell count at diagnosis (*p*=0.4492) (**Supplementary Figure 6A**). Regarding the FAB classification, M4 subtype had the highest median HAVCR2 expression level (9.40) and M0 subtype had the lowest (7.84). A significant difference was observed among all FAB subtypes (*p*=0.0090) (**Figure 4A**). In addition, patients with CBF translocations had significantly higher mRNA expression level of HAVCR2 as compared to those without CBF translocations (median [range], 9.81 [8.21-10.40] versus 8.69 [3.14-11.49], *p*<0.0001) (**Figure 4B**). With respect to ELN risk category, the low risk group (median [range], 9.63 [3.14-11.49]) had significantly higher HAVCR2 expression level than the intermediate (8.75 [3.83-10.60]) (*p*=0.0302) and high (8.39 [4.84-10.19]) (*p*<0.0001) risk groups, however no significant difference was observed between the intermediate and high risk groups (*p*=0.1500) (**Figure 4C**).

In order to evaluate the impact of HAVCR2 expression levels on clinical outcomes, AML patients were first divided into low and high expression groups based on the median HAVCR2 expression level, and the probability of 5-year OS was not significantly different between two groups (low versus high, 24.95% [95% CI 14.86%-36.39%] versus 24.54% [95% CI 13.59%-37.20%], *p*=0.6660) (**Figure 4D**). Furthermore, patients with low, intermediate and high ELN risks were divided into low and high TIM-3 expression subgroups, respectively, and the OS did not differ significantly between two subgroups in any ELN risk group (**Supplementary Figures 6B–D**).

## Discussion

In the present study, we simultaneously assessed the TIM-3 expression level of leukemic blasts and T lymphocytes in the bone marrow of *de novo* AML patients using flow cytometry, and found that the TIM-3 expression level of leukemic blasts correlated positively with T lymphocytes. Besides, TIM-3 expression of leukemic blasts correlated positively with the percentage of peripheral CD8^+^ T lymphocytes. When analyzed with clinical data, higher TIM-3 expression of leukemic blasts was shown to significantly correlate with the presence of CBF translocations but not with the survival of patients, and similar results were also obtained with TCGA data.

TIM-3 is a member of the TIM family of immunoregulatory proteins. It was originally discovered in T cells and has been considered as an important checkpoint receptor. So far, four ligands, which are Gal-9, phosphatidylserine, high-mobility group protein B1 and carcinoembryonic antigen-related cell adhesion molecule 1 (CEACAM1), have been identified to bind TIM-3. Among them, Gal-9 and CEACAM1 are involved in TIM-3 mediating the inhibition and apoptosis of T cells (7). TIM-3 is a glycoprotein containing both N-linked and O-linked glycans, and glycosylation is required for Gal-9 binding (21, 22).

Besides T cells, TIM-3 also expresses on AML cells, rather than normal hematopoietic stem cells, and is involved in the self-renewal of LSCs. Autocrine Gal-9 binds to TIM-3 and subsequently activates NF-κB and β-catenin signaling, promoting LSC self-renewal (11). In addition, TIM-3 is currently thought to play a crucial role in immune escapes of AML cells. TIM-3, together with its ligand Gal-9, can be released from AML cell surface in a free soluble form through proteolysis, and impair the killing activity of NK cells and IL-2 production of T cells (12). TIM-3 also have a higher expression, together with other checkpoint receptors, like PD-1, on T cells of AML patients as compared to healthy adults (23), and this is believed to be associated with severe T cell exhaustion and disease progression (10).

Our data showed that TIM-3 expression levels on leukemic blasts varied among AML patients, which were in accordance with those of Kikushige et al. and Xu et al. (15, 24) Due to the important role of TIM-3 in AML, it would be intriguing to determine whether varied expression levels of TIM-3 on AML cells influence the immune status and prognosis of AML patients.

Our results revealed a correlation of TIM-3 expression of leukemic blasts with TIM-3 expression of both CD8^+^ and CD4^+^ T lymphocytes and the proportion of CD8^+^ T lymphocytes, indicating that TIM-3 expression of leukemic blasts might alter adaptive immunity of AML patients. TIM-3 on T lymphocytes is generally considered as a marker of T cell exhaustion, especially when coexpressed with other checkpoint receptors (25). For example, TIM-3^+^PD-1^+^CD8^+^ T cells in mice with advanced AML exhibited severe exhausted phenotype as defined by failure to produce IL-2, tumor necrosis factor and interferon-γ, and combined targeting of TIM-3 and PD-1 pathways was more effective in controlling the leukemia burden (10). TIM-3 was also reported to coexpress with two other checkpoint receptors LAG-3 and PD-1 on severe exhausted tumor-infiltrating lymphocytes of patients with glioblastoma (26). Therefore, we consider that our data support the notion that TIM-3 onleukemic blasts can promote an inhibitory immune environment and facilitate the immune escape of AML cells (27). In the future, it would be worthwhile to assess, together with the TIM-3 expression, the expression of other checkpoint receptors and perform the function assays of lymphocytes in AML patients, in order to further determine the effect of TIM-3 expression of leukemic blasts on adaptive immunity.

The relation of TIM-3 with AML prognosis is currently controversial. In our study, the TIM-3 expression level of leukemic blasts was neither correlated with the CR after the first induction chemotherapy nor the survival of AML patients. This data are contradictory to those of Darwish et al., Kamal et al. and Xu et al. In studies of Darwish et al. and Kamal et al., all AML subtypes including M3 were enrolled. M3 patients usually have good prognosis and hardly express TIM-3 on their leukemic blasts. This might partly explain different conclusions they got from those of our study. In the study of Xu et al., AML patients with high TIM-3 expression level achieved a higher CR rate than patients with low TIM-3 expression level (91% versus 67%, *p*=0.01), while the survival data were not reported. Our data, which include both treatment response after the induction chemotherapy and the survival of patients, are supposed to be more reliable. Moreover, TCGA data of 157 non-M3 AML patients were subsequently analyzed and showed that TIM-3 expression was not associated with the OS of patients, either in the whole cohort or in any ELN risk group, consistent with results of our cohort. Interestingly, Wang et al. recently reported that high TIM-3 expression levels of T and NK cells as a whole and CD34^+^CD38^-^ cells were significantly associated with a high 2-year cumulative incidence of relapse in t(8;21) AML patients (28). Unfortunately, it is impossible to confirm this finding with our data, as only few patients in our cohort have t(8;21). Further studies are needed to verify this finding and explore the underlying molecular mechanisms.

It should be noted that we assessed TIM-3 expression level on the surface of leukemic blasts in our cohort, while in TCGA data, TIM-3 mRNA expression level in the peripheral blood samples was used for analysis. In the peripheral blood of AML patients, TIM-3 expresses not only in leukemic blasts, but also in multiple other cell types, e.g. T lymphocytes, NK cells, monocytes, etc. However, leukemic blasts are the main cell population (average percentage of 39.6% in non-M3 AML patients of TCGA data), and our data also showed that TIM-3 expression of T cells, another big cell population, correlated positively with that of leukemic blasts, therefore TIM-3 mRNA expression in the peripheral blood is supposed to represent that of leukemic blasts.

CBF translocations include t(8;21)(q22;q22) and inv (16)(p13q22)/t(16;16)(p13;q22), which lead to the formation of fusion genes AML1/ETO and CBFβ/MYH11, respectively. These fusion genes disrupt the signaling of heterodimeric CBF complex in a dominant negative manner, resulting in impaired hematopoietic differentiation (29). AML with CBF translocations accounts for approximately 15% of adult AML cases and is stratified as favorable risk (30). Our results showed that CBF translocations were associated with higher TIM-3 expression on the surface of leukemic blasts. Similar results have been reported by Jan et al. and Xu et al., and it is thought that mutations in CBF may either directly regulate TIM-3 transcription or arrest leukemic cells in a stage of differentiation with high TIM-3 expression (15, 31). Silva et al. reported that latrophilin1/protein kinase C/mammalian target of rapamycin pathway was involved in the expression of TIM-3 and its ligand Gal-9 in AML blasts, but all their experiments were performed in the cell line of THP-1, which carries KMT2A-MLLT3 fusion gene rather than CBF translocations (12). Further studies are needed to elucidate the molecular mechanism by which CBF translocations control TIM-3 expression.

Since AML with CBF translocations are considered as favorable risk, it seems that higher TIM-3 expression should be associated with good prognosis, which is not the case in our study. When we analyzed the data carefully, we found that other low-risk genetic alterations, e.g. NPM1 mutation with wild-type FLT3-ITD, usually had low TIM-3 expression level, and some intermediate-risk AML patients also had high TIM-3 expression level. Eventually, TIM-3 expression level was not related with the clinical outcome of AML patients.

Currently, TIM-3 is considered as a potential target for the treatment of myeloid malignancies. Since the autocrine Gal-9 binding to TIM-3 on the surface of leukemic blasts drives the self-renewal of AML stem cells (11), it is a potential therapeutic strategy to block TIM-3 using anti-TIM-3 mAbs. Several monoclonal antibodies (mAbs) against TIM-3, e.g. MBG453 (NCT03066648) and SHR-1702 (NCT04443751), are investigated in ongoing clinical trials and their clinical efficacy is unknown (32, 33). According to our study, it seems that TIM-3 expression on leukemic blasts does not influence the outcome of AML patients, or traditional chemotherapy can overcome the adverse impact of TIM-3 on outcomes of AML patients. Therefore, we should be cautious to expect that anti-TIM-3 mAbs further improve the clinical outcome of AML patients. However, anti-TIM-3 mAbs bound to leukemic blasts may facilitate antibody-dependent cellular phagocytosis by myeloid cells/macrophages, and they may also block TIM-3 on lymphocytes and other immune cells and potentially enhance the immune response against leukemic blasts (32). Therefore, it remains intriguing to see whether anti-TIM-3 mAbs have a therapeutic effect on myeloid malignancies.

Our study has limitations. First, the cohort size is relatively small, and further studies with larger sample size are still required to confirm these findings. Second, immunophenotyping of multiple checkpoint receptors, other than TIM-3, and function assays of lymphocytes were not performed in our study. These experiments are needed to assess adaptive immune status. Third, flow cytometry can only detect the protein of TIM-3 on the cell surface. It would be better to simultaneously assess HAVCR2 mRNA level using RT-PCR to confirm the results. Forth, western blot should be performed to assess the glycosylation status of TIM-3 in these AML samples, as glycosylation may influence the function of TIM-3, like the binding with Gal-9 (21).

In conclusion, TIM-3 expression of AML blasts correlated positively with TIM-3 expression of T lymphocytes and the proportion of CD8^+^ T lymphocyte, indicating TIM-3 might alter adaptive immunity of AML patients. In addition, TIM-3 expression of AML blasts correlated with CBF translocations rather than the survival of patients. Therefore, TIM-3 might not be a good biomarker for non-M3 AML prognosis under current treatment modalities.

## Data Availability Statement

The raw data supporting the conclusions of this article will be made available by the authors, without undue reservation.

## Ethics Statement

The studies involving human participants were reviewed and approved by The Ethical Committee of the First Affiliated Hospital of Anhui Medical University. The ethics committee waived the requirement of written informed consent for participation.

## Author Contributions

MY and YW conceived and designed the study. JH and LX performed the experiments, analyzed the data and wrote the manuscript. JH and XY performed TCGA data collection and analysis. JH, LX, ZH, XL, JD, ZW, LL, MR, ZL, XC, XWC, JN, JG, QL, QZ and RX performed clinical sample and data collection. All authors contributed to the article and approved the final version.

## Funding

The study was supported by Research Fund of Anhui Institute of Translational Medicine (Grant No. 2021zhyx-C70), Clinical Medicine Discipline Construction Project of Anhui Medical University (Grant No. 2020lcxk034) and Young Scholar Program of The First Affiliated Hospital of Anhui Medical University (Grant No. 2019kj20).

## Conflict of Interest

The authors declare that the research was conducted in the absence of any commercial or financial relationships that could be construed as a potential conflict of interest.

## Publisher’s Note

All claims expressed in this article are solely those of the authors and do not necessarily represent those of their affiliated organizations, or those of the publisher, the editors and the reviewers. Any product that may be evaluated in this article, or claim that may be made by its manufacturer, is not guaranteed or endorsed by the publisher.
