# Supplementary material for: TIM-3 Expression Level on AML Blasts Correlates With Presence of Core Binding Factor Translocations Rather Than Clinical Outcomes
Source: Front Oncol. 2022 Apr 14;12:879471. doi: 10.3389/fonc.2022.879471 (PMC9046698; doi:10.3389/fonc.2022.879471)
Supplement: Supplementary file 1 [file DataSheet_1.pdf]

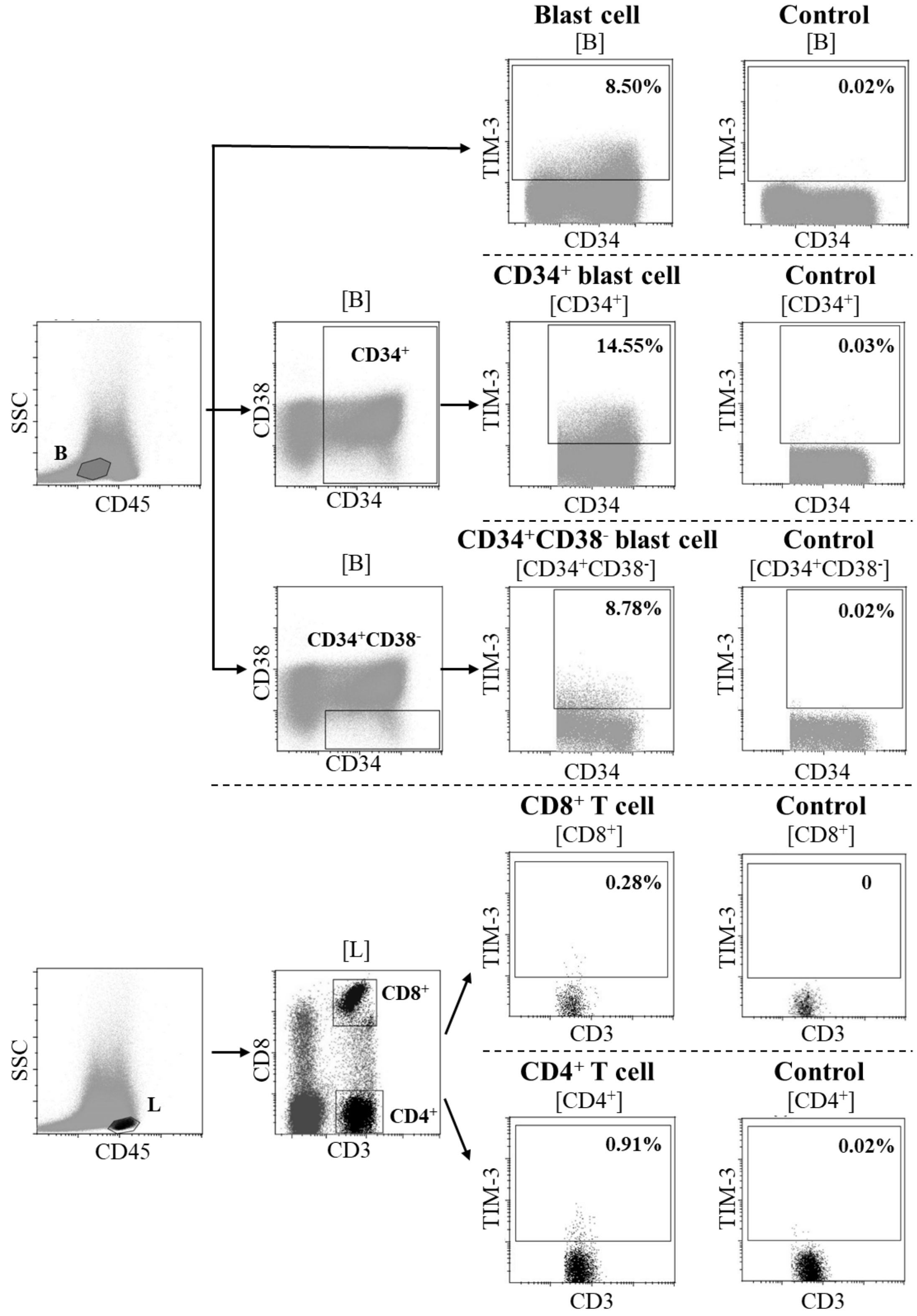

Supplementary Figure 1. The gating strategy of TIM-3 expression of leukemic blasts and T cells in the bone marrow of AML patients.

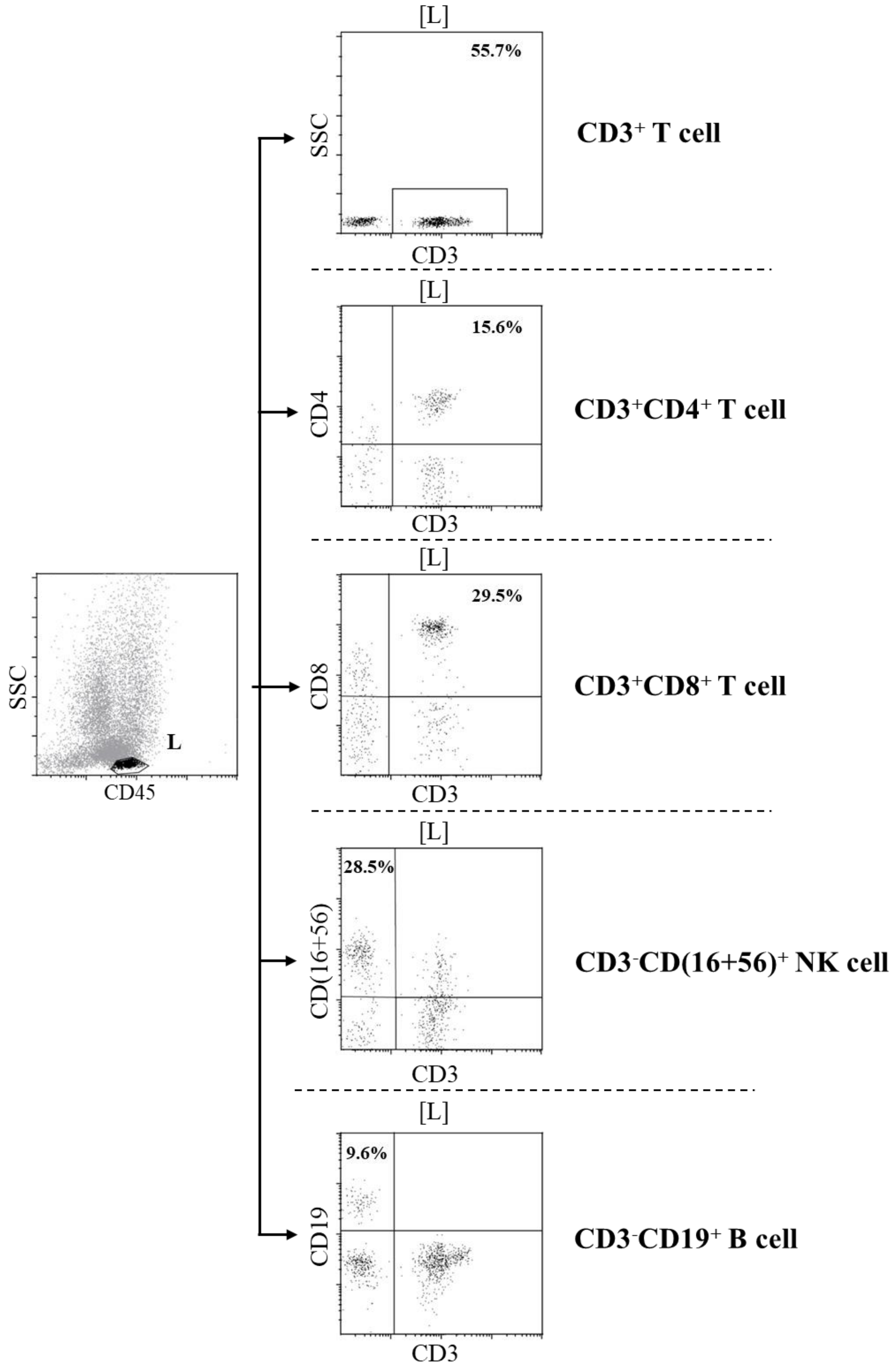

Supplementary Figure 2. The gating strategy of lymphocyte subtypes in the peripheral blood of AML patients.

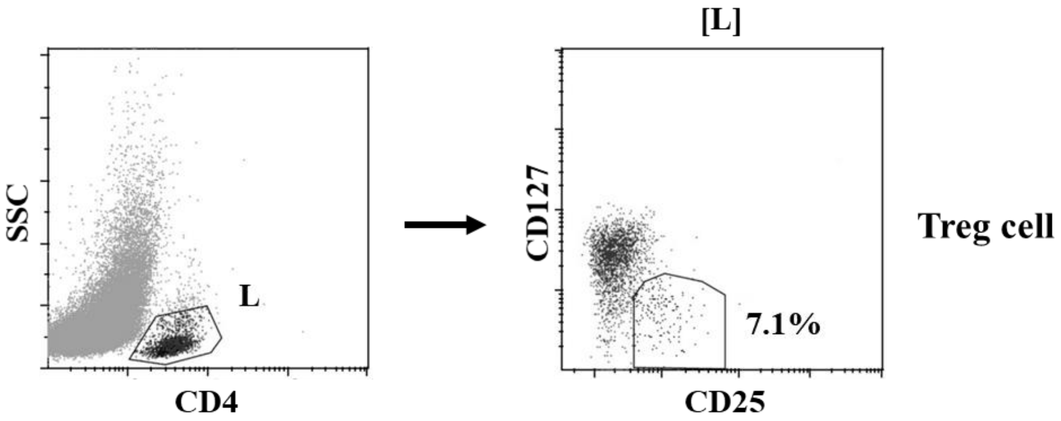

Supplementary Figure 3. The gating strategy of $CD4^{+}CD25^{+}CD127^{-}$ Treg cells in the peripheral blood of AML patients.

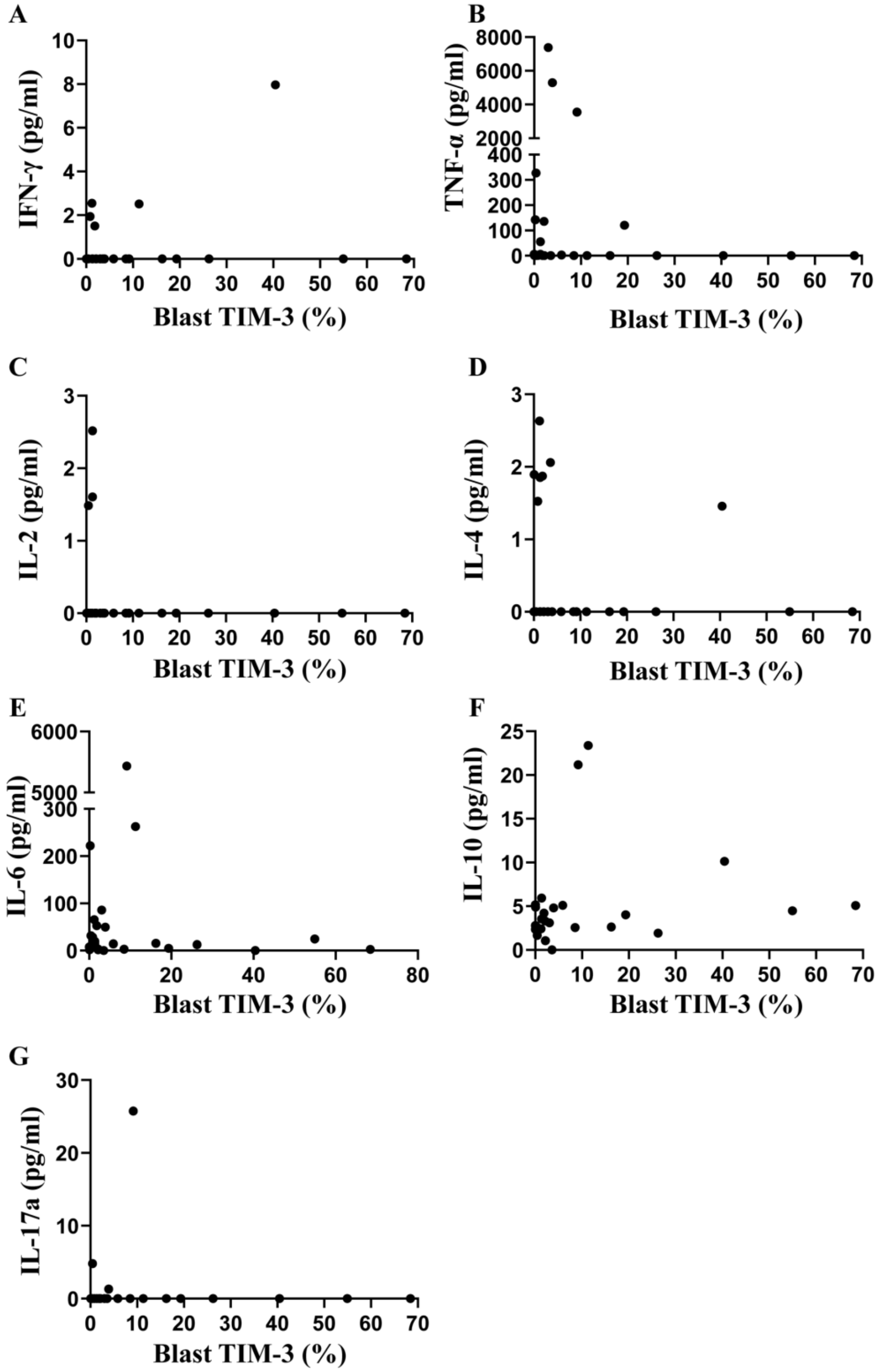

Supplementary Figure 4. Associations of TIM-3 expression level of leukemic blasts with plasma levels of Th1/Th2/Th17 cytokines in AML patients. Th1/Th2/Th17 cytokines, including IFN-γ (A), TNF-α (B), IL-2 (C), IL-4 (D), IL-6 (E), IL-10 (F) and IL-17a (G), were assessed in 27 out of 34 AML patients using cytometric bead array cytokine kit. Th, T helper cell; IFN-γ, interferon-gamma; TNF-α, tumor necrosis factor-alpha; IL, interleukin.

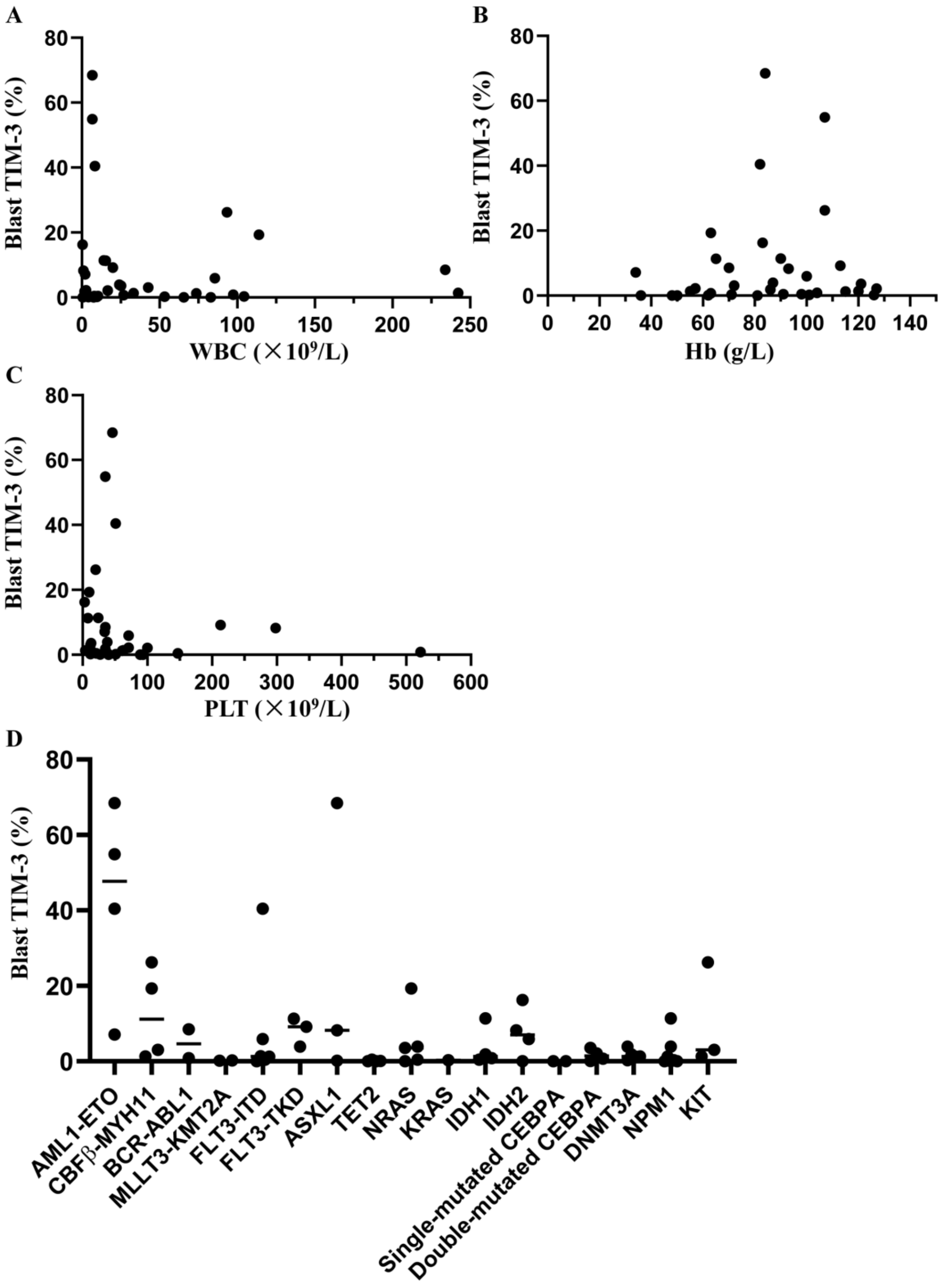

Supplementary Figure 5. Associations of TIM-3 expression level of leukemic blasts with levels of white blood cell (A), hemoglobin (B) and platelet (C) and various genetic alterations (D).

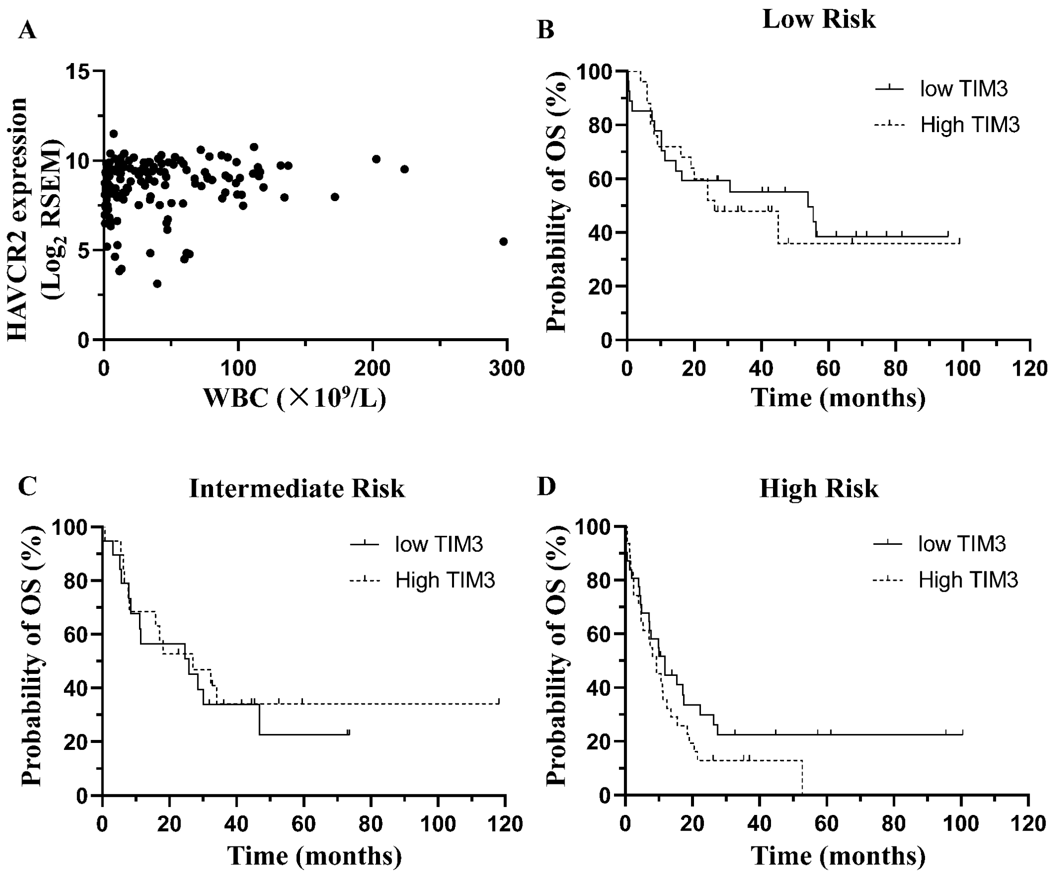

Supplementary Figure 6. TCGA dataset: Associations of HAVCR2 mRNA expression level (RNA Seq V2 RSEM) in non-M3 AML patients with several clinical parameters. Association of HAVCR2 expression level with peripheral white blood cell numbers of patients is shown in (A). Patients in low, intermediate and high ELN risk groups were divided into low and high HAVCR2 expression subgroups, respectively, based on the median HAVCR2 expression level. Probabilities of OS of two subgroups in low (B), intermediate (C) and high (D) risk groups were shown.
